# Potential Effects of Climate Change on the Water Level, Flora and Macro-fauna of a Large Neotropical Wetland

**DOI:** 10.1371/journal.pone.0067787

**Published:** 2013-07-09

**Authors:** Bárbara Úbeda, Adrian S. Di Giacomo, Juan José Neiff, Steven A. Loiselle, Alicia S. Guadalupe Poi, José Ángel Gálvez, Silvina Casco, Andrés Cózar

**Affiliations:** 1 Department of Biology, Faculty of Marine and Environmental Science, Campus CEI·MAR, University of Cádiz, Puerto Real, Spain; 2 Instituto de Ecología, Genética y Evolución de Buenos Aires (CONICET-UBA), Facultad de Ciencias Exactas y Naturales, Universidad de Buenos Aires, Buenos Aires, Argentina; 3 Centro de Ecología Aplicada del Litoral, CECOAL, Consejo Nacional de Investigaciones Científicas y Técnicas, CONICET, Corrientes, Argentina; 4 University of Siena, Siena, Italy; University of Sydney, Australia

## Abstract

Possible consequences of climate change in one of the world’s largest wetlands (Ibera, Argentina) were analysed using a multi-scale approach. Climate projections coupled to hydrological models were used to analyse variability in wetland water level throughout the current century. Two potential scenarios of greenhouse gas emissions were explored, both resulting in an increase in the inter-annual fluctuations of the water level. In the scenario with higher emissions, projections also showed a long-term negative trend in water-level. To explore the possible response of biota to such water-level changes, species-area relationships of flora and aerial censuses of macro-fauna were analysed during an extraordinary dry period. Plant species richness at the basin scale was found to be highly resistant to hydrological changes, as the large dimension of the wetland acts to buffer against the water-level variations. However, local diversity decreased significantly with low water levels, leading to the loss of ecosystem resilience to additional stressors. The analysis of macro-fauna populations suggested that wetland provides refuge, in low water periods, for the animals with high dispersal ability (aquatic and migratory birds). On the contrary, the abundance of animals with low dispersal ability (mainly herbivorous species) was negatively impacted in low water periods, probably because they are required to search for alternative resources beyond the wetland borders. This period of resource scarcity was also related to increased mortality of large mammals (e.g. marsh deer) around water bodies with high anthropogenic enrichment and cyanobacteria dominance. The synergy between recurrent climatic fluctuations and additional stressors (i.e. biological invasions, eutrophication) presents an important challenge to the conservation of neotropical wetlands in the coming decades.

## Introduction

Freshwater wetlands can be strongly affected by climate change as their spatial and environmental characteristics are closely dependent on the water balance of the basin [1]-[2]-[3]. Modelling analyses suggest that climate effects on surface water balances will vary widely globally, both in magnitude and direction [2].

The Neotropics include several of the world’s largest freshwater wetlands, associated with the vast floodplains of large South American rivers (e.g. Amazon, Orinoco, Parana, Paraguay). Their flora and fauna show outstanding species richness with an important fraction of endemic species [4]-[5]-[6]. Long-term trends of water level in wetlands may have special relevance for species richness due to their impact on ecosystem size and habitat diversity [7]. Short-term fluctuations of the water level determine the ecosystem stability, which influences the species diversity and composition [8]-[9].

The Ibera wetland is one of the largest neotropical wetlands (13,000 km^2^) and a world reservoir of biodiversity [10]-[11]-[12]. The Ibera wetland originated as floodplain of the Parana River, with the progressive displacement of the Parana course at the end of the Pleistocene disconnecting the floodplain from the river. The modification of the ancient fluvial course contributed to habitat heterogeneity and species richness [13]. The deepest depressions resulted in permanent open water bodies, while the low hills became the sites of permanent forests. A variety of wetland environments can be found in relation to the inundation level (e.g. occasionally, seasonally or quasi-permanently flooded), resulting in a mosaic of isolated forests, grasslands, marshes, swamps and dispersed lakes supporting a rich wildlife.

Ecological studies integrating spatial, temporal and trophic scales provide an innovative basis for decision making in large conservation areas [14]. Such approach becomes particularly relevant when exploring the impacts of climate change, as it can reveal indications of system change or broad-scale interrelationships that are difficult to detect with more partial analyses. However, the costs of large-scale monitoring programs hinder the application of this approach in the complex (i.e. species rich) systems characterizing many tropical areas [15]. Valuable data may not be efficiently explored due to a lack of integration over wider (spatial, temporal or trophic) scales. Therefore, the success of this approach depends on the combination of a comprehensive efficient monitoring plan with effective strategies for information treatment [15].

In the present study, we apply an integrated multi-scale approach to analyse the possible ecological consequences of climate change in the Ibera wetland. Climate impacts on water level were assessed by coupling hydrological models and climate projections under two scenarios of greenhouse gas emissions. Water-level simulations and historical data were used to analyse the long-term trend and the magnitude of the fluctuations at annual and inter-annual scales. Between 2005 and 2008, frequent droughts led to significant variability in wetland water levels. The performance of extensive samplings of flora and macro-fauna during this period provided the opportunity to explore the potential response of the wetland biota to the future hydrological scenarios linked to the climate change.

## Methods

### Study Area

**Figure 1:**
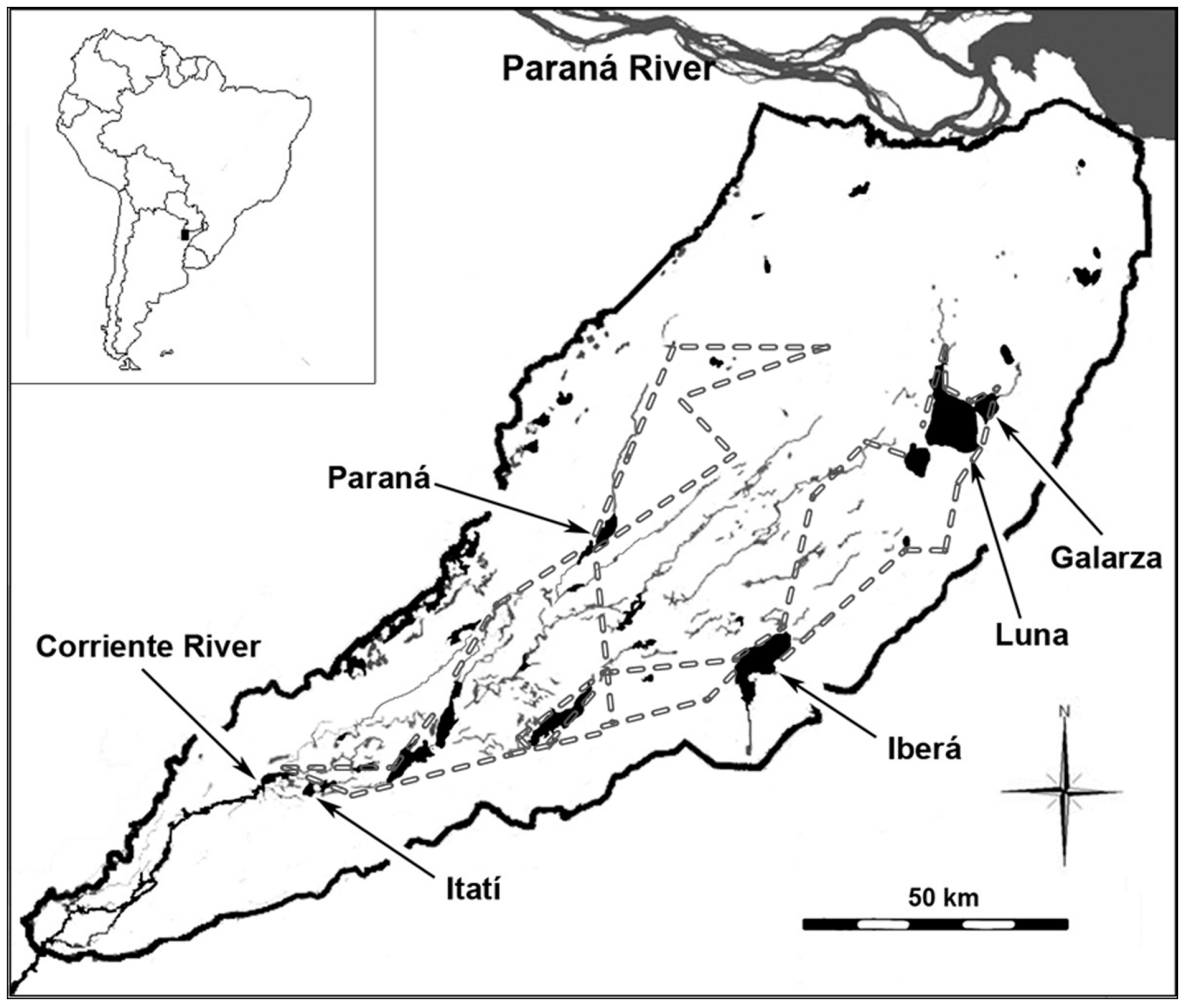
The Ibera wetland. Black thick lines indicate the wetland border. The locations of the water bodies of the present study are indicated. The flight route of the macro-fauna aerial censuses is shown with a broken line.

The Ibera wetland is a large subtropical wetland located in northeastern Argentina, between 27° 36′-28° 57′ S and 58° 00′–57° 30′ W. A majority of the wetland has been protected since1983. Ibera is primarily fed by direct rainfall, while river inflow is insignificant. Limited drainage occurs through Corriente River (Fig. 1), due to the very flat slope (gradient around 1∶10,000) and the large amount of vegetation accumulated in the basin. There are eleven large (>5 km^2^) open water bodies with a mean depth of around 2.5 m [16]. Most of the permanently or quasi-permanently flooded areas are densely vegetated and often include areas of floating wetlands (locally termed as *embalsados*). These have originated from the interlacing of floating plants and the accumulation of organic matter from plant decay [17]. In the mature state, the *embalsados* may reach a thickness of 4 m and support high vegetation biomass, even small forested areas.

### Hydrological Analysis

The hydrological analysis was conducted on two open water bodies (*Laguna Iberá* and *Laguna Galarza*) located in two main depressions of the Ibera plain (Fig. 1). The DYRESM model (http://www.cwr.uwa.edu.au) was used to simulate the water-level variability in these water bodies. DYRESM is a one-dimensional hydrodynamic model which has been often used to simulate temperature and water level in lakes and reservoirs. The water body is modelled as a series of horizontal lagrangian layers, with uniform properties and variable thickness based on changes in lake depth. Meteorological data are used to determine incoming energy due to short-wave radiation and surface heat fluxes due to evaporation, sensible heat, long-wave radiation and wind stress.

Hourly data of solar radiation (short wave and long wave), air temperature, vapour pressure, wind and rain were obtained between February 1999 and November 2000 at shore positioned meteorological stations. Simultaneous water level and water temperature data were obtained in hydrological stations in the offshore areas of the lakes. Twenty field surveys also provided data of water level and temperature.

Data collected in 1999 were used for hydrological model calibration while data collected in 2000 were used for model validation [18]. Model simulations from 2010 to 2100 were derived from monthly climate projections generated by the third generation coupled global climate model (CGCM, http://www.cccma.ec.gc.ca). CGCM is a fully coupled atmospheric-ocean global circulation model used in the IPCC’s Fourth Assessment Report [19]. We assessed both a conservative and a non-conservative emission scenarios [19]. The conservative scenario (B2) is associated to a mean increase in air temperature of 1.4–3.8°C and in global CO_2_ concentration of 600 p.p.m. by volume (p.p.m.v.) for 2100. The non-conservative scenario (A2) results in air temperature increase of 2.0–5.4°C and CO_2_ increase of 800 p.p.m.v. The regional projection of each meteorological variable was transformed into anomalies in relation to the average conditions modelled for 1999–2000. These trends in relative variability were then applied to the average meteorological conditions measured in1999–2000 in *Laguna Iberá* and *Laguna Galarza.*

Historical measurements of water level in the two study lakes were compiled from the *Subsecretaría de Recursos Hídricos* of Argentina Government (SRH, www.hidricosargentina.gov.ar) and international research projects [20]. A 70-year-long daily series of water level (1929–2008), with a 7-year break (1960–1968), was available for *Laguna Iberá*. A smaller data series was available for Laguna Galarza (1968–1979).

All water-level data were standardized as mean water levels by using the hypsographic curves of the two lacustrine depressions studied [21]-[22]. Changes in the historical and projected water levels were analyzed at different time scales. The slope of the linear regression of the water-level time series was used to evaluate the long-term trend. Water-level fluctuations at inter-annual scale were assessed from the coefficient of variation (CV) of the monthly water level by decade. At annual scale, water-level fluctuations were assessed from the seasonal amplitude of the water level (annual maximum – annual minimum).

### Vegetation Diversity

Extensive field surveys were performed during the spring-summer of 1976–77 and 2007–08. The 1976–77 period was representative of the most common hydrological conditions in Ibera, while the 2007–08 period was characterized by abnormally low water input. Water drainage through Corriente River averaged 255±125 m^3^ s^−1^ in 1976–77, and 73±47 m^3^ s^−1^ in 2007–08 (SRH, www.hidricosargentina.gov.ar). Plant species composition was analyzed in 20-m^2^ plots across the coasts of five of the main water bodies of the wetland (*Iberá, Galarza, Luna, Paraná* and the floodplain of Corriente River; Fig. 1). The number of target plots was 36 for 1976–77 period and 137 for 2007–08.

Taxonomic inventories in the target plots showed the diversity of hydrophilic species, including vascular aquatic macrophytes, macroalgae, mosses and the vegetation growing on the *embalsados*. A detailed description of species and life-forms composition and the differences between water bodies is shown in reference [23]. The vegetation diversity at basin scale was explored using a species–area relationship (*SAR*) using the *EstimateS* software package [24].

The relationship between the number of species (*S*) and the sampling area (*A*) was characterized by fitting the species-area data. Traditionally, the most commonly used functions are convex (not asymptotic) curves, particularly the exponential and the power functions. However, the shape of *SAR* is generally sigmoidal and reaches an upper asymptote at a specific area in large isolated ecosystems such as Ibera wetland [25]. Among the candidate models to fit the *SARs* [26], the Chapman function showed the best agreement with our results:
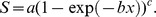
where *a* is the asymptote, and *b* and *c* are parameters related to the curvature of the function. We use a common upper asymptote (*a*) of 680 hydrophilic species (including those growing on *embalsados*) [11]. One of the two curvature parameters was fixed to avoid excessive differences in the shape of the *SARs* curves for the two hydrological scenarios studied. This prevented the crossing of the *SARs* curves, which implies that the scenario with higher species number at small wetland areas is predicted to have lower richness at large areas. We fixed the c parameter at 1/4, which showed the best overall regression for the data from our two scenarios. Regression analyses were performed through STATISTICA 10.0 software (StatSoft, Inc.).

### Animal Abundance

Aerial counts have been shown as a suitable method to monitor abundance of macro-fauna populations (e.g. large mammals, caymans) in Ibera [27]-[28] and similar open landscapes in southwestern Brazil [29]-[30]-[31]. In the present study, aerial censuses were performed from an Aero Boero-180 airplane in February 2007 and February 2008 (austral summer).The flight route (600 km), altitude (100 m) and speed (150 km h^−1^) were standardized in both surveys (Fig. 1). During the flight, we recorded the animals observed within a fixed 200 m strip. The sampled area covered around 3% of the wetland area, including the main wetland habitats. Animals were classified into 3 classes of domestic macro-fauna (sheep, horses and cows) and 11 classes of wild macro-fauna (Table 1). Buffalos were introduced in Ibera, but escaped buffalos live wild. All these fauna groups are relatively abundant in Ibera, although two species show particular conservation interest at a global scale. Marsh deer (*Blastocerus dichotomus)* is categorized as vulnerable and greater rhea (*Rhea americana*) as near-threatened [32]. Counts were in tens of individuals when they occurred at high densities (e.g. waterfowl, cattle). A total of 5,418 individuals were counted in the two surveys.

**Table 1:** Groups of wild fauna monitored during the aerial surveys in Ibera wetland.

Group	Species or family	Class	IUCN Category	Main diet items
Greater rhea	*Rhea Americana*	Aves	Near-threatened	Plants
Capybara	*Hydrochoerus hydrochaeris*	Mammalia	Least concern	Plants
Cayman	*Caiman yacaré* and *C. latirostris*	Reptile	Least concern	Fishes
Marsh deer	*Blastocerus dichotomus*	Mammalia	Vulnerable	Plants
Duck	Anatidae	Aves	Least concern	Plants
Water buffalo	*Bubalus bubalis*	Mammalia	Non-native	Plants
Southern screamer	*Chauna torquata*	Aves	Least concern	Plants
Storks and herons	Ciconiidae and Ardeidae	Aves	Least concern	Fishes
Ibises	Threskiornitidae	Aves	Least concern	Invertebrates
Shorebirds	Charadriidae and Scolopacidae	Aves	Least concern	Invertebrates
Neotropic cormorant	*Phalacrocorax olivaceus*	Aves	Least concern	Fishes

IUCN: International Union for Conservation of Nature.

The period between 2005 and 2008 was characterized by frequent droughts and generally below average water levels. Nevertheless, significant yearly differences were evident throughout this period. The summer of 2008 was characterised by particularly low rainfall water. Rainfall in the summer of 2008 (106 mm) was less than one quarter of that measured in the summer of 2007 (442 mm; SRH, www.hidricosargentina.gov.ar). We must note that the dry summer of 2008 was also preceded by a particularly cold winter and a weakened annual cycle of winds (SRH; data from Corrientes city). Mean temperatures in the winters before 2008 and 2007 samplings were 13.9±1.4°C and 18.0±1.0°C respectively (16.7±1.0°C for the 1997–2006 period). Mean wind speed was 2.1±1.0 m s^−1^ in the 12 months preceding February 2008 and 3.7±1.3 m s^−1^ in the 12 months preceding February 2007 (3.6±1.2 for the 1997–2006 period). The climate anomalies that occurred in the annual cycle from autumn 2007 to summer 2008 were related to a major La Niña event.

Based on the significant climatic differences associated to the two sampling periods (summer 2008 and summer 2007), we determined the yearly variation in the faunal censuses to discuss how the drought and other climatic anomalies could be involved in the changes observed. The comparison of absolute counts was avoided due to the differences in the detectability of the fauna groups [33]. Results were analyzed as relative change within each group.

## Results

**Figure 2:**
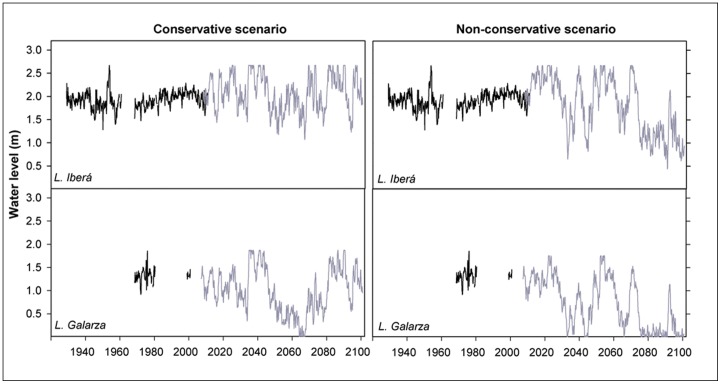
Historical and predicted monthly variability of the water level in Ibera wetland. Monthly water level projections of *Laguna Iberá* (top) and *Laguna Galarza* (bottom) in the conservative (left) and non-conservative (right) climate scenarios are shown with the historic water level in black and the water level projection in grey.

The validation of the hydrological model showed a strong agreement between model outputs and field data of temperature (R = 0.95, p<0.0001, n = 244) and water level (R = 0.99, p<0.0001, n = 972; Figure S1). Model simulations indicated a progressive warming of the lake waters, at a rate of 0.02°C y^−1^ in the conservative (B2) scenario to 0.03°C y^−1^ in the non-conservative (A2) scenario (Figure S2). Using the B2 climate projection, simulations showed a relatively limited change in the long-term averaged water level in *Laguna Iberá* (Table 2, Fig.2). In the A2 scenario, a moderate decline in the mean water level was found in the second half of the century (−0.32 m in relation to the mean historic level). A more consistent fall of the water level was predicted for *Laguna Galarza* where both scenarios led to a decline, −0.13 m and −0.47 m in the first and second half of century for the B2 scenario, and −0.41 m and −0.77 m in the first and second half of century for the A2 scenario (Table 2, Fig. 2).

**Table 2:** Characteristics of the water level in *Laguna Iberá* and *Laguna Galarza* for different (measured and modelled) time periods and climate scenarios.

		Historicdata	Non-conservative (A2 ) scenario		Conservative (B2) scenario		
Laguna Iberá		1929–2010(n = 839)	2010–2050	2051–2100	2010–2050		2051–2100
	Mean water level (m)	1.92±0.17	2.06±0.13	1.60±0.12	2.13±0.12		2.00±0.14
	Seasonal amplitude (m)	0.39±0.17	0.42±0.23	0.43±0.21	0.39±0.25		0.47±0.19
	CV at inter-annual scale (%)	8.79±5.55	19.67±11.57	24.03±11.88	16.07±1.80		16.60±4.93
	Long-term trend (cm y−1)*	+0.43±0.13	+0.00±0.16	−0.58±0.10	+0.46±0.11		+0.12±0.06
Laguna Galarza		1968–2010 (n = 150)					
	Mean water level (m)	1.35±0.21	0.92±0.09	0.55±0.08	1.17±0.40	0.85±0.50	
	Seasonal amplitude (m)	0.31±0.21	0.32±0.17	0.29±0.16	0.34±0.24	0.38±0.17	
	CV at inter-annual scale (%)	7.51±4.69	41.46±28.78	80.00±40.91	27.77±7.83	38.48±24.36	
	Long-term trend (cm y−1)*	+0.16±0.22	−1.03±0.19	−1.00±0.11	−0.11±0.16	−0.27±0.11	

* The long-term trends are calculated in both lakes from 1968 until the end of the period referred.

Seasonal variability was predicted to increase moderately (<8 cm). However, water-level fluctuations at an inter-annual scale showed significant increases (Table 2). Simulations included frequent periods of extremely high and low waters in both climatic scenarios (Fig. 2). It should also be noted that the models show temporary dry periods for Laguna Galarza, mainly in the A2 scenario.

**Figure 3:**
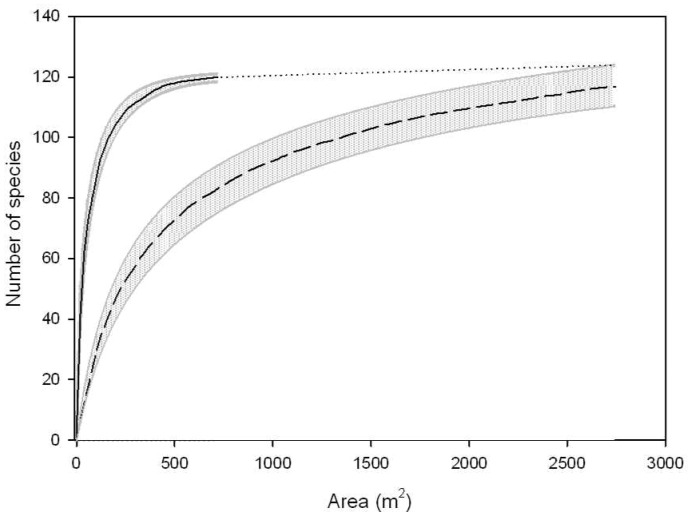
Species-area relationships for the vegetation in Ibera wetland. Species-area relationships in 2007–08 (dry period, black dashed line) and 1976–77 (black solid line) produced by randomizing samples (100 runs) with *EstimateS* [24]. The 95% confidence intervals are shadowed and the dotted line is an extrapolation of *SAR* for 1976–77.

The taxonomic analyses of flora showed a total of 161 species during the two study periods, 120 species in 1976–77 and 117 species in 2007–08. *SARs* of both periods had an asymptotic behaviour, although with different shapes. The largest differences were found in the initial slope of the curves (Fig. 3). At larger spatial scales (>2,000 m^2^), the species richness was similar in both study periods.

**Figure 4:**
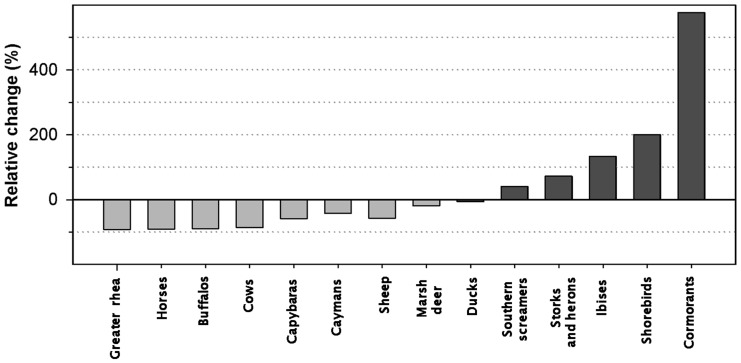
Variation in the aerial census of different groups of macro-fauna in 2007 and 2008. Percentages indicate the change in the censuses in summer 2008 (dry period) in relation to summer 2007. Positive values indicate a higher count in summer 2008. Bars of the bird groups with high dispersal ability are filled with dark grey.

The response of macro-fauna to the dry summer was highly variable, with both positive and negative responses (Fig. 4). Nevertheless, the response pattern was consistent with dispersal ability and feeding requirements. Animals with low dispersal ability tended to decrease, whereas the animals with high dispersal ability increased. The number of flying birds, particularly piscivorous (cormorants, storks and herons) and benthivorous (ibises and shorebirds), drastically increased in the dry summer. Herbivorous birds such as screamers or ducks showed a moderate increase or remained practically invariable. Conversely, the census of flightless birds (greater rhea) radically decreased in the dry summer. Among the mammals, marsh deer decreased by 19% and capybaras decreased by 58%.

## Discussion

In the conservative scenario, the hydrological models predict a slight decrease of the long-term water level in the wetland, significant only in the shallower *Laguna Galarza*. In the non-conservative scenario the negative trend in water level was generalized and more pronounced, with frequent episodes in which *Laguna Galarza* completely dried out. These results suggest a reduction in the extension of Ibera wetland. Such losses of wetland area have occurred in the past. Cartographic data from the end of XIX century (Francisco M. T. Fouilliand 1891) and data from the 1911 scientific expedition by the Scientific Society of Argentina show a smaller wetland area was present at the beginning of the past century.

The total richness of hydrophilic plants (*S_max_*) in Ibera was earlier reported as 680 species [11]. In the present work, the area-related inventory of hydrophilic species was made on 3,460 m^2^, less than 0.0001% of the wetland area. In contrast, we identified 23.7% of the total number of hydrophilic species. In a large and isolated wetland such as Ibera, *S_max_* is probably reached in a relatively small fraction of the flooded area. The species-area curve should reach an upper asymptote (*S_max_*) in a relatively low spatial area (*A_Smax_*) [25]. Therefore, it is not expected that an expansion or reduction of the wetland area will lead to a significant increase or decrease of *S_max_* if the variety of habitat types is maintained. However, we found that water-level decline in 2007–08 affected the species richness at local scale (<2,000 m^2^; Fig. 3), which could result in a variation of *A_Smax_*. The fitting of species-area data to Chapman functions (R = 0.9727, p<0.0001 for 1976–77; R = 0.9637, p<0.0001 for 2007–08) was used to extrapolate *A_Smax_* in each sampling period. *A_Smax_* was larger in 2007–08 but considerably lower than the total flooded area, varying from 5·10^6^ in 1976–77 to 3·10^7^ m^2^ in 2007–08, which represent between 0.1 and 0.3% of the wetland area. These estimates are sensitive to the function used to extrapolate *SAR*, although the low values of *A_Smax_* in relation to the wetland dimension are consistent. This analysis suggests that the vastness of Ibera acts as a buffer against losses in species richness following contractions of the wetland area. Consequently, flora species richness in Ibera is likely to be highly resistant to climate-driven hydrological changes. In fact, even though climate variability in the region has been significant the past [34]-[35], paleolimnologic studies of pollen composition reported that the species diversity has not significantly changed during the last three thousand years [36].

The yearly variation in the fauna censuses may be due to changes in population dynamics (i.e. net mortality), migratory flows or animal behaviour. In the dry summer of 2008, considerable increases in the number of birds with high dispersal ability were observed. Much of these birds use Ibera for nesting, dispersing later throughout the region (cormorants, storks, herons and ibises) or travelling to other latitudes (migratory shorebirds) [37]. The presence of aquatic and migratory birds in Ibera during the drought of 2008 is likely related to the scarceness of alternative foraging areas in the region. Thus, Ibera would act as a refuge against the abnormally dry periods. Additionally, piscivorous and benthivorous bird guilds can take advantage of the transitory low-water episodes, when fish and benthic resources become more accessible in patchy areas within the wetland. Resource concentration during low water episodes was reported to be advantageous to wetland caymans [38]-[39]. However, our cayman counts are probably influenced by changes in aerial detectability related to the population grouping in small areas of high resource abundance [30].

The drought of the summer of 2008 led to an extensive decrease of vegetation production and biomass in the Ibera wetland. The result was that the presence of (herbivorous) domestic macro-fauna in the wetland dramatically decreased. Three aerial counts of cows performed in Ibera before summer 2008 were quite stable (1,054±77; Di Giacomo, unpublished data), but the count fell to 153 in summer 2008. Domestic macro-fauna are frequently moved outward or sold during periods of resource scarceness in the wetland. The general reduction in the censuses of wild herbivores with limited dispersal ability (capybara, marsh deer, buffalo and greater rhea) is probably related to a change in the animal behaviour as result of the need for additional resources. In the aerial counts, many of these animals were observed around the lakes or in the wetland borders. Studies in the nearby Pantanal wetland showed that marsh deer congregated around the permanent water bodies during the annual dry season [29]-[31]. Aquatic plants represent an important resource for the marsh deer in this riverine floodplain [40]. Nevertheless, grasses are a staple food for all the herbivores studied, including marsh deer. We suggest that the incursion into the upland grasslands surrounding the wetland is a common foraging strategy for many herbivores during periods of food scarcity. This behaviour would imply that the protection and accessibility of the grasslands beyond the wetland borders are key for mitigating the effects of the climate fluctuations on herbivores with limiting dispersal ability.

The reduction in the numbers of capybaras and marsh deer can also be related to an elevated mortality in the previous winter, especially in the endangered marsh deer population. In an aerial survey performed in winter 2007, 10.2% of the total marsh deer observed were dead individuals. A total of 30 marsh deer deaths were recorded through aerial and terrestrial surveys during the winter of 2007 in Ibera. While, past high mortality events of marsh deer were associated to cattle-borne diseases [27], an analysis of the sick and dead deer in winter 2007 showed the absence of any infectious diseases and a low ectoparasite burden. Marsh deer showed emaciation, diarrhea and submandibular edema [41]. Blood analyses showed high urea concentrations, and feces and gastrointestinal contents had high quantities of hematophagous parasites (*Haemonchus contortus*). Marsh deer mortality was directly related to gastrointestinal parasitism and a resulting anemia, but the mechanisms that induced the gastrointestinal infestation or the cause of the high urea concentrations in the sampled blood remained unclear [41].

Interestingly, two thirds of the records of marsh deer deaths in winter 2007 were located in the area around *Laguna Iberá*. This lake is connected to rice cultivations located on the wetland border and support the largest human settlement in the wetland. Using a 1997–2001 dataset of remote-sensed water properties in the twenty-six largest wetland lakes, the area around *Laguna Iberá* was specifically identified as disturbed [16]. Field samplings performed during 2007 in six large water bodies (*Iberá, Galarza, Luna, Paraná, Itatí* and Corriente River) found the highest phytoplankton biomass concentrations and the largest dominance of cyanobacteria in *Laguna Iberá* [42]. In this lake, phytoplankton abundance has increased five-fold and cyanobacteria percentage (from 60 to 90% of total) doubled in relation to historical conditions [43].The high abundance of cyanobacteria observed in *Laguna Iberá* in 2007 is probably related to the progressive nutrient loading of the lake and the weakening of the winds during the 2007–08 La-Niña cycle. Such conditions provide particularly favourable conditions for the cyanobacteria development [44]-[45].

Unlike other lakes in Ibera, cyanobacteria in *Laguna Iberá* were widely dominated by *Cylindrospermopsis raciborskii* [42]. Channels and small ponds in the vicinity of *Laguna Ibera* also became dominated by this species. *C.raciborskii* is one of the most notorious for its ability to produce a wide variety of potent cyanotoxins, with disruptive effects on organs such as the kidney [46]-[47]. It has been implicated in mortality episodes of cattle [48], with human effects limited to sickness [49]-[50].

Cyanobacteria blooms in Ibera lakes extend from summer to autumn, while the winter determines the seasonal decline of the blooms [51]-[42]. The cellular content of cyanotoxins in *C. raciborskii* increases during the bloom decline [47]. Additionally, lower temperatures (as those occurred in winter 2007) reinforce the stability of the cyanotoxins in water [52]. Therefore, the winter is the most plausible period to observe cyanobacteria-induced intoxication in Ibera. Winter fish kills are sporadically observed in local areas of *Laguna Iberá*, and other minor episodes of deer deaths have been also noticed in winter by the park rangers [41]. There are several indications of possible links between the marsh deer mortality and the toxic algal blooms. Given the ecological implications of this hypothesis, specific studies should be designed to assess the influence of algal toxins on the deer deaths.

Besides the negative long-term trend in wetland water level, the most relevant feature of the hydrological projections was the increase in water-level fluctuations. This arises from an increased frequency of extreme climate events predicted by the global circulation models [19]. Wetlands are transitional environments, and their plant species are commonly adapted to environmental fluctuations [53]. Our analysis suggests that plant species richness at the basin scale is highly resistant to hydrological changes, although local diversity was considerably affected (Fig. 3). Losses of local diversity linked to hydrological fluctuations were reported in other wetlands (e.g. [54]-[55]-[56]). Following disturbances, ecosystems generally move from a mature state of high diversity to an earlier stage of succession [8]. Theoretical and empirical evidence emphasizes the importance of the timing of the disturbance, as the recurrent reduction in ecosystem maturity leads to arrested successions, prolonged periods of low diversity and long-term loss of resilience [8]-[9]. In this regard, we must note the significantly lower vegetal species richness in the active floodplain of the Parana River with respect to that of the Ibera wetland [23]. The main environmental differences between these two wetlands are the amplitude and frequency of the water-level fluctuations, which are considerably higher and less regular in the Parana floodplain.

The loss of local diversity in the flora implies a lower variety of resources (e.g. seeds, fruits, nectar) throughout the year with the largest impacts on fauna with small home ranges (around 2,000 m^2^ or lower; mainly invertebrates). Nevertheless, small-scale perturbations could be also transferred to animals with larger home ranges [57]. In the Pantanal wetland, the bird species richness (including a major fraction of birds with small home ranges) was found to decrease with the habitat instability [58].

The flora and fauna of Ibera has experienced strong climate disturbances in the past. However, the expected increase in climate fluctuations will occur in combination with unprecedented levels of anthropogenic stressors. For example, climate anomalies can combine with the eutrophication trends to increase the magnitude of the cyanobacteria blooms [45] and, consequently, the potential exposure of the wild fauna to cyanotoxins. The confirmation of the influence of the algal toxic blooms on the mortality of marsh deer would be a meaningful example. Biological invasions also merit special attention. Naturalization of ornamental plants (*Cladium jamaicensis*), forestry tree species (*Pinus elliottii*), and domestic animals (*Sus scrofa, Bubalus bubalis*) have become common in Ibera [59]-[60]. Climatic fluctuations are well-known factors mediating the establishment and expansion of invasive alien species [61]. Therefore, the positive interaction between climate change and anthropogenic stressors could become the Achilles’ heel of the Ibera macrosystem. To counteract this synergism, regional policy makers should increase efforts to reduce the nutrient loading of the wetland and control the biological invasions.

### Considerations for the Integrated Monitoring of Large Wetlands

In the present study, we have integrated historical data gathering, long-term hydrodynamic modelling and large-scale surveys of flora and macro-fauna to analyse the potential effects of climate change on the Ibera wetland..

The hydrological models used (i.e. DYRESM) provided a high level of accuracy. However, it should be noted that forecasting the climate impacts on hydrological conditions is subject to uncertainty due to its dependence on human behaviour, which is inherently uncertain. Nevertheless, the climate-coupled hydrological projections in different human-behaviour scenarios serve to identify possible trends in the hydrological regime of wetlands.

The response of the biota to climate change is clearly more complex due to the plethora of indirect interactions and feedbacks between biotic and abiotic elements in the ecosystem. In this regard, the analysis of extreme climatic events appears to be a pragmatic way to undertake this task. Regional *SARs* were found to be a suitable method for assessing the basin-scale state of the vegetation diversity. Likewise, aerial censuses allowed for a large scale assessment of diverse groups of macro-fauna. The implementation of these methods in long-term monitoring programs would improve, with a moderate economic investment, our capacity to understand the effects of climatic and anthropogenic forcing on biota of large wetlands.

## Supporting Information

Figure S1 **Calibration and validation of the hydrological models.** Water temperature in *Laguna Iberá* (a) and *Laguna Galarza* (c). Water level in *Laguna Iberá* (b) and *Laguna Galarza* (d). Model calibration was performed with the data collected in 1999 and validation with the data collected in 2000. Model outputs are shown as lines and field data as circles. Breaks in model simulations are due to lack of meteorological data.
(TIF)

Figure S2 **Predicted trends of temperature in lakes of Ibera.** Water temperature projections correspond to *Laguna Iberá* (a) and *Laguna Galarza* (b). Orange line limit is derived from the non conservative (A2) scenario, while blue line limit is derived from the conservative (B2) scenario.
(TIF)
