# Supplementary figures and images for: Potential Effects of Climate Change on the Water Level, Flora and Macro-fauna of a Large Neotropical Wetland
Source: PLoS One. 2013 Jul 9;8(7):e67787. doi: 10.1371/journal.pone.0067787 (PMC3706436; doi:10.1371/journal.pone.0067787)

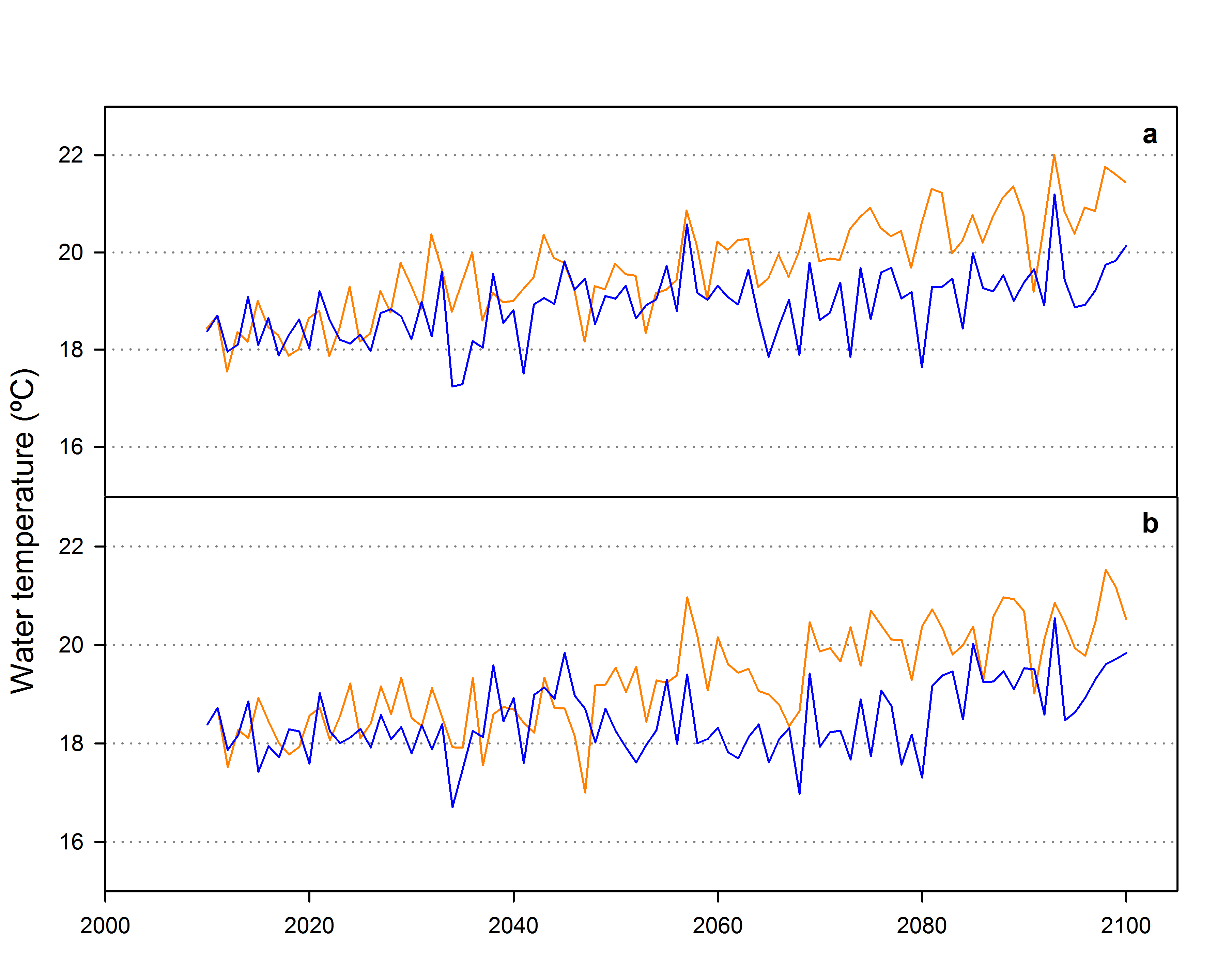

Supplement: Figure S2 — Predicted trends of temperature in lakes of Ibera. Water temperature projections correspond to Laguna Iberá (a) and Laguna Galarza (b). Orange line limit is derived from the non conservative (A2) scenario, while blue line limit is derived from the conservative (B2) scenario. (TIF) [file pone.0067787.s002.tif]
